# Differences in the BAL proteome after *Klebsiella pneumoniae *infection in wild type and SP-A-/- mice

**DOI:** 10.1186/1477-5956-8-34

**Published:** 2010-06-17

**Authors:** Mehboob Ali, Todd M Umstead, Rizwanul Haque, Anatoly N Mikerov, Willard M Freeman, Joanna Floros, David S Phelps

**Affiliations:** 1 Penn State Center for Host defense, Inflammation, and Lung Disease (CHILD) Research, Hershey, PA 17033, USA; 2 Department of Pediatrics, The Pennsylvania State University College of Medicine, Hershey, PA 17033, USA; 3 Department of Pharmacology, The Pennsylvania State University College of Medicine, Hershey, PA 17033, USA; 4 Department of Obstetrics and Gynecology, The Pennsylvania State University College of Medicine, Hershey, PA 17033, USA

## Abstract

**Background:**

Surfactant protein-A (SP-A) has been shown to play a variety of roles related to lung host defense function. Mice lacking SP-A are more susceptible to infection than wild type C57BL/6 mice. We studied bronchoalveolar lavage (BAL) protein expression in wild type and SP-A-/- mice infected with *Klebsiella pneumoniae *by 2D-DIGE.

**Methods:**

Mice were infected intratracheally with *K. pneumoniae *and after 4 and 24 hours they were subject to BAL. Cell-free BAL was analyzed by 2D-DIGE on two-dimensional gels with pH ranges of 4-7 and 7-11. Under baseline conditions and at 4 and 24 hr post-infection BAL was compared between untreated and infected wild type and SP-A-/- mice. Sixty proteins identified by mass spectrometry were categorized as host defense, redox regulation, and protein metabolism/modification.

**Results:**

We found: 1) ~75% of 32 host defense proteins were lower in uninfected SP-A-/- vs wild type, suggesting increased susceptibility to infection or oxidative injury; 2) At 4 hr post-infection > 2/3 of identified proteins were higher in SP-A-/- than wild type mice, almost the exact opposite of untreated mice; 3) At 24 hr post-infection some proteins continued increasing, but many returned to baseline; 4) In infected wild type mice significant changes occurred in 13 of 60 proteins, with 12 of 13 increasing, vs on 4 significant changes in SP-A-/- mice. Infection response patterns between strains demonstrated both commonalities and differences. In several cases changes between 4 and 24 hr followed different patterns between strains.

**Conclusions:**

These indicate that SP-A plays a key role in regulating the BAL proteome, functioning indirectly to regulate lung host defense function, possibly via the macrophage. In the absence of SP-A baseline levels of many host defense molecules are lower. However, many of these indirect deficits in SP-A-/- mice are rapidly compensated for during infection, indicating that SP-A also has a direct role on host defense against *K. pneumoniae *that may be instrumental in determining clinical course.

## Introduction

Pulmonary surfactant is a lipoprotein complex essential for normal lung function. The protein component of pulmonary surfactant consists of hydrophobic and hydrophilic proteins including surfactant protein A (SP-A). SP-A has been shown to play a crucial role in innate immune function in the lung. Among the reported functions of SP-A in this regard are: enhancing the clearance of pathogens by acting as an opsonin [1,2], regulating the production of cell surface antigens and inflammatory mediator expression by immune cells [3,4], participating in the development of dendritic cells [5], regulating reactive oxidant production [6,7], and others [1,8]. Mice lacking SP-A have been shown to have increased susceptibility to a variety of infectious agents [9-11] and were found to have increased mortality after infection with *Klebsiella pneumoniae *as compared to wild-type mice [12]. The mechanism(s) by which SP-A exerts these effects are not well understood. In some cases the observed function appears to be directly attributable to SP-A via its well-documented ability to enhance phagocytosis of some pathogens [1,2], but because SP-A can regulate expression of various regulatory molecules, including some cytokines, it is also likely that at least some of these functions are indirect effects of SP-A. Comparing the bronchoalveolar lavages (BAL) from C57BL/6 wild type (WT) and SP-A-/- mice on the same genetic background [13] demonstrated that SP-A had the ability to influence a diverse collection of proteins and also demonstrated an exaggerated response to oxidative stress (following an acute ozone exposure) in SP-A-/- mice, suggesting altered regulation in the absence of SP-A.

*K. pneumoniae*, a gram-negative bacteria and a member of the Enterobacteriaceae family has long been recognized as a possible cause of community-acquired pneumonia in individuals with impaired pulmonary defenses [14]. We [12,15] and others [16,17] have employed a mouse model of *K. pneumoniae *pneumonia to study the mechanisms responsible for host defense against this pathogen and the implications of infection. These studies have demonstrated increased severity when infection follows an acute oxidative stress, as a consequence of hyperoxia or ozone exposure [12,15,16]. Furthermore, reports of increased susceptibility to *K. pneumoniae *infection in mice lacking SP-A [12], lysozyme [18], and β 2-microglobulin [19] indicate that the host defense against this pathogen may be multifactorial.

In order to explore the impact of *K. pneumoniae *infection on the BAL proteome in WT and SP-A-/- mice we employed two-dimensional difference gel electrophoresis (2D-DIGE), an unbiased discovery proteomics technique [20-22] for quantitation of proteins, coupled with Matrix Assisted Laser Desorption Ionization-Time-of-Flight/Time-of-Flight (MALDI-ToF/ToF) tandem mass spectrometry for identification of proteins. Using these techniques we can simultaneously analyze large numbers of proteins in the BAL. Two-dimensional gel-based techniques, including 2D-DIGE, have been used to study the BAL proteome in lung disease [23-25] and in various experimental systems [13,26-29]. These techniques may also have the potential to implicate pathways that may not have been previously suspected to play a role in these systems. We recently employed a similar approach to examine age-related changes in the rat BAL proteome [29] and to characterize ozone-induced changes in the mouse BAL proteome [13]. As in our previous studies [13,29] we used the PANTHER database and the published literature to assign many of the proteins identified to three major categories: host defense function (DEF); redox regulation (RED); and protein metabolism and modification (PMM).

The rationale for the present study is based on the two following hypotheses: 1) The absence of SP-A compromises host defense against *K. pneumoniae *increasing susceptibility to infection and the severity of disease. 2) SP-A influences the levels of expression of other proteins with host defense function, thereby predisposing SP-A-/- mice to infection. To test these hypotheses we investigated differences in BAL protein expression between C57BL/6 WT and SP-A-/- mice before infection and in response to *K. pneumoniae *infection with a discovery proteomics approach. Simultaneous analyses of dozens of proteins and their isoforms in biological samples can help in the identification of pathways and proteins involved in the host response to *K. pneumoniae *infection. The type of unbiased approach used in this study does not depend on previously published studies and may be instrumental in generating specific novel hypotheses involving proteins and pathways that may not have been previously implicated in the processes being studied.

In this study we compared the BAL proteomes of untreated WT and SP-A-/- mice infected with *K. pneumoniae *for 4 and 24 hr and studied the resulting changes in the BAL proteome using 2D-DIGE, coupled with MALDI-ToF/ToF for protein identification.

## Materials and methods

### Animals

This study was conducted with pathogen-free male WT and SP-A-/- male mice on the C57BL/6 genetic background. WT mice were obtained from Jackson Laboratories (Bar Harbor, ME) and housed under standard environmental conditions prior to the experiment. Breeder pairs of SP-A-/- mice were obtained from Dr. Samuel Hawgood at the University of California, San Francisco and were bred and raised under specific pathogen-free conditions in a barrier facility at the Penn State College of Medicine. The SP-A-/- mice and sentinel mice housed in the same room showed no evidence of respiratory pathogens. The Institutional Animal Care and Use Committee at the Penn State College of Medicine approved this study.

A total of 24, 11-12 week old (20-25 g) C57BL/6 WT and SP-A-/- mice were used in the present study. These were divided into six groups with 4 animals per group: 1) WT control that did not receive any treatment; 2) WT exposed to *K. pneumoniae *for 4 hr; 3) WT exposed to *K. pneumoniae *for 24 hr; 4) SP-A-/- control that did not receive any treatment; 5) SP-A-/- exposed to *K. pneumoniae *for 4 hr; 6) SP-A-/- exposed to *K. pneumoniae *for 24 hr.

### Bacteria

*K. pneumoniae *bacteria (ATCC 43816) were obtained from the American Tissue Culture Collection (Rockville, MD). Bacteria were inoculated into 50 ml of tryptic soy broth (TSB) in 250 ml flasks for 18 hr at 37°C (stationary phase), with shaking at 120 rpm (Incubator Series 25, New Brunswick Scientific Co., Edison, NJ). The bacterial suspension was diluted in TSB to obtain an OD_660 _of 0.4 and then 200 μl of this diluted bacterial suspension was added to 50 ml of TSB for 3 hr to reach mid-log phase of growth (OD_660 _~0.4, corresponding to ~2 × 10^8 ^CFU/ml), where bacteria are most virulent. Bacteria were placed on ice to stop their growth and then serially diluted in PBS to obtain ~9 × 10^3 ^CFU/ml. For infection, 50 μl of this suspension (~450 CFU/mouse) were used as in our previous studies [12,15].

### Infection of mice with *K. pneumoniae*

Animals were anesthetized with an intramuscular injection of a mixture of Ketamine HCl (Ketaject, Phoenix Pharmaceuticals Inc., St. Joseph, MO) and Xylazine (XYLA-JECT, Phoenix Pharmaceuticals Inc., St. Joseph, MO). The trachea was surgically exposed and ~450 CFU/mouse were inoculated intratracheally in 50 μl of PBS. Skin incisions were closed with 7 mm wound clips. Mice infected in this way are not in apparent respiratory distress and mortality during the first 48 hours is rare. As described in our previous studies, the dose administered was an LD_50 _for WT male mice in survival studies, with most mortality occurring between 3 and 5 days [15].

### BAL fluid

The lungs of the anesthetized mice were subjected to BAL at intervals of 4 and 24 hr following infection. BAL fluid was obtained by instilling saline into the lungs through a tracheal cannula using a volume equal to 80% of lung vital capacity (3 × with 0.5 ml of 0.9% NaCl) for a total of 1.5 ml. Total BAL fluid recovery was approximately 90% of the instilled volume and did not differ significantly between the experimental groups and controls. The BAL fluid was centrifuged (150 × g, 10 min, 4°C) and cell-free supernatant was frozen at -80°C for subsequent proteomic studies.

### BAL protein assessment

Protein concentrations were determined using the Bio-Rad Protein Assay (Bio-Rad, Hercules, CA) before and after protein precipitation. For precipitation one volume of ice cold 100% TCA was added to four volumes of protein sample, which were mixed and incubated overnight at 4°C. Following overnight incubation, samples were centrifuged (15,000 × g, 15 min, 4°C) and the protein pellets washed with 250 μl of chilled acetone (-20°C), centrifuged again, resuspended in a minimum volume of standard cell lysis buffer (30 mM Tris HCl, 2 M thiourea, 7 M urea, 4% CHAPS, 8.5). The concentration of protein was brought to 1 mg/ml for CyDye labeling.

### One-dimensional gel electrophoresis

Protein (12.5 μg) from each sample was subjected to gel electrophoresis using ExcelGel 12.5% SDS-PAGE separating gels with a 5% stacking gel. Gels were run at 20 mA for 30 min and then for 1 hour at 50 mA and then silver stained (SilverQuest™ Silver Staining Kit, Invitrogen, Carlsbad, CA).

### Protein identification from one-dimensional gel electrophoresis

Bands of interest were excised from 1D-gels after silver staining and processed for MALDI-ToF/ToF mass spectrometry (4800 Proteomic Analyzer Applied Biosystems, Foster City, CA) as described below.

### 2D-DIGE labeling (minimal labeling) and electrophoresis for 2D-DIGE

Information about the 2D-DIGE study is provided in a form that complies with the most recent version. http://www.psidev.info/miape/MIAPE_GE_1_4.pdf of Minimum Information About a Proteomics Experiment-Gel Electrophoresis (MIAPE-GE) standards currently under development by the Human Proteome Organization Proteomics Standards Initiative (see Additional File 1). Two sets of samples from each group were prepared for minimal labeling with CyDyes. Samples from each group were randomly assigned to Cy3 or Cy5 to ensure no dye-based artifacts in quantitation. A 25 μg aliquot of BAL protein from each sample was labeled with either Cy3 or Cy5 (200 picomoles). A normalization pool was created by combining equal amounts of protein from every sample (24 samples) and an aliquot of the pool was labeled with Cy2 (200 picomoles/25 μg). Equal amounts (25 μg) of Cy3-labeled sample, Cy5-labeled sample, and Cy2-labeled pool samples were mixed and applied to each gel. The use of a normalization pool is advantageous as this serves as an internal standardization tool for all gels/samples under study, and thus normalizes any quantitative differences due to gel-to-gel variability. An equal volume of 2 × sample buffer (2 M thiourea, 7 M urea, 2% IPG buffer (pH 4-7 or pH 7-11 nonlinear (NL)) and 1.2% DeStreak reagent) was added to all samples to give a final volume of 150 μl. The 24 cm pH 4-7 and pH 7-11NL gradient Immobiline DryStrips (GE Healthcare) were rehydrated for 16 hr with 450 μl of rehydration buffer (DeStreak™ Rehydration Solution containing 0.5% IPG buffer (pH 4-7 or pH 7-11). Proteins were subjected to isoelectric focusing on rehydrated strips using the Ettan IPGphor 3 cup loading manifold (GE Healthcare) following manufacturer's instruction at 20°C and under mineral oil to prevent evaporation. Proteins were focused by using the following voltages and times: 3 hr at 300 V (step and hold); 7 hr at 1000 V (gradient); 4 hr at 8000 V (gradient); 4 hr at 8000 V (step and hold). After isoelectric focusing the IEF strips were equilibrated in equilibration solution-1 (50 mM Tris HCl, 6 M urea, 30% glycerol, 2% sodium dodecyl sulphate (SDS), 0.5% dithiothreitol) and equilibration solution-2 (50 mM Tris HCl, 6 M urea, 30% glycerol, 2% SDS, 4.5% iodoacetamide) for 15 min, respectively, and then applied to 10-14% gradient polyacrylamide gels (26 cm-w × 20 cm-h × 1 mm thick), sealed with 0.5% low melting point agarose containing bromophenol blue in a buffer of 1 × Tris/glycine/SDS buffer (25 mM Tris, 192 mM glycine, 0.1% (W/V) SDS, pH 8.3) and run for 30 min at 5 W/gel and then for 6-7 hr at 14 W/gel at 20°C using the Ettan DALTtwelve system (GE Healthcare) for separation of proteins on the basis of molecular weight.

For preparative (picking) gels an aliquot of 350 μg of sample was diluted with an equal volume of 2 × sample buffer (2 M thiourea, 7 M urea, 2% IPG buffer (pH 4-7 or pH 7-11) and 1.2% DeStreak reagent) and then brought up to a volume of 450 μl with rehydration buffer (DeStreak™ Rehydration Solution and 0.5% IPG buffer (pH 4-7 or pH 7-11)). Proteins were focused using the following voltages and times: 14 hr at 0 V (passive rehydration); 6 hr at 30 V (active rehydration); 3 hr at 300 V (step and hold); 3 hr at 600 V (gradient); 3 hr at 1000 V (gradient); 3 hr at 8000 V (gradient); 4 hr at 8000 V (step and hold). Each of the strips was equilibrated as described above and applied to a 10-14% gradient polyacrylamide gels (26 cm-w × 20 cm-h × 1 mm thick). For the preparative picking gel a single plate for each gel plate sandwich was treated with Bind-Silane solution (80% ethanol, 0.02% glacial acetic acid, 0.001% Bind-Silane) and had reference marker stickers placed on them. After the completion of electrophoresis, the plates that had not been silane-treated were removed from the sandwich and the gels were fixed overnight with 30% ethanol, 7.5% glacial acetic acid. The preparative picking gels were then stained with Deep Purple Total Protein Stain (GE, Healthcare) for 2 hr.

### Gel scanning, image analysis, and statistics

Information about the acquisition and processing of data from the 2D-DIGE studies are provided in the form that complies with the most recent version of the guidelines established for Minimum Information about a Proteomics Experiment - Gel Informatics (MIAPE-GI) currently under development by the Human Proteome Organization Proteomics Standards Initiative. http://www.psidev.info/files/miape-gi-v1.pdf (see Additional File 2). All two-dimensional gels were imaged on a Typhoon 9400 fluorescent imager (GE Healthcare) at a resolution of 100 μm. Photomultiplier tube voltages were individually set for each of the three colored lasers to ensure maximum, linear signals. The same voltages were used for all the gels. The DIGE gels were imaged at three different wavelengths (Cy2: 520 nm; Cy3: 580 nm; Cy5: 670 nm) and the Deep Purple Total Protein Stain-stained gels were imaged at 100 μm with a separate filter (610 nm).

Gel images were imported into the Progenesis SameSpots v2.0 program (Nonlinear Dynamics) for analysis and their quality assessed by the program. Image quality control with Progenesis SameSpots v2.0 involves checking images for bit depth, color, manipulation prior to analysis, proper file type, saturation, low dynamic range, and stretched contrast. A reference gel with minimum distortion and streaks was selected from the Cy 2 gels. Gel alignment was conducted automatically and then checked manually to ensure correct alignment. Spot detection and spot matching across all the gels were conducted automatically, and then spot matching was checked and manually edited to ensure correct matching. Data from all the spots included in analysis were transported to Progenesis PG240 module of the Progenesis SameSpots v2.0 software for further analysis. Statistical analysis was performed by t-test to confirm the level of significance among various groups.

For identified proteins having multiple isoforms, the normalized volumes of all isoforms of a given protein were added together and statistical analysis was performed on the totals using Microsoft Excel.

### Protein identification by mass spectrometry

For identification of spots, protein spots were picked from picking gels using a robot-directed spot picker (Ettan Spot Picker, GE Healthcare). The spots selected for picking were determined on the basis of differential expression from the 2D-DIGE analyses. The picker head was calibrated using the reference stickers placed on the preparative picking gel and the gels were picked and gel plugs placed in a bar-coded 96 well plate. All gel plugs were washed twice with 200 μl of 200 mM ammonium bicarbonate, 40% acetonitrile for 30 min at 37°C and dehydrated one time with 75% acetonitrile for 20 min followed by air drying. The protein was then digested with 20 μl of 0.02 μg/μl trypsin (Trypsin, proteomics grade, Sigma, St. Louis, MO) overnight at 37°C. Fifty μl 50% acetonitrile, 0.1% trifluoroacetic acid (TFA), was next added to each plug and incubated for 30 min at 37°C. Digested proteins/peptides were then transferred to 96-well extraction plates, dried by speed vac (Vaccufuge™ Concentrator, Eppendorf AG, Hamburg, Germany) and resuspended in 10 μl 0.5% TFA. Extracted proteins/peptides were desalted and concentrated using C_18 _ZipTips (Millipore Corporation, Billerica, MA). Tips were wetted with 10 μl of 100% acetonitrile and equilibrated with 10 μl 0.1% TFA pH < 4. Samples were then drawn into ZipTip columns by aspirating for 7 cycles and then washed twice with 10 μl 0.1% TFA. Peptides were then eluted from the column with 5 μl of 50% acetonitrile, 0.1% TFA.

Peptides were then analyzed by MALDI-ToF/ToF mass spectrometry (4800 Proteomic Analyzer Applied Biosystems, Foster City, CA) in the Mass Spectrometry Core at the Penn State University College of Medicine. A total of 3 μl of ZipTip cleaned samples (1 μl at a time) was applied onto a 384-well MALDI plate (Opti-TOF™ 384 Well Insert, Applied Biosystems) and then 0.7 μl of 2 mg/ml ACH cinnamic acid in 60:40 (acetonitrile: water) was then spotted on each well containing peptide. All 13-calibration wells on the MALDI plate were spotted with (1:12 diluted) 4700 calibrant. Autolytic trypsin peptides were also used to internally calibrate the spectra to an accuracy of 20 ppm. Using the GPS Explorer 3.0.software (Applied Biosystems), the MS and MS/MS data were submitted to the MASCOT search engine using the NCBI non-redundant and SwissProt databases and mouse taxonomy for identification. MASCOT confidence interval scores of > 95% were considered as positive protein identification. The PANTHER database and the scientific literature were used to assign molecular function and biological process to each identified protein. We assigned the identified proteins to several broad functional classes including: a) host defense proteins (DEF); b) proteins involved in regulating redox balance (RED); and c) proteins involved in protein metabolism and modification (PMM). It should be noted that some proteins are in more than one functional group. This classification scheme, which we have used in other studies [13,29] was more inclusive than relying solely on the biological function classification provided by PANTHER and similar gene ontology databases. We also used the Ingenuity Pathway Analysis program (Ingenuity Systems, Redwood City, CA) to gain additional insight into the functional significance of the observed changes.

## Results and Discussion

We investigated the hypothesis that SP-A plays a role in the host defense of mouse lungs at baseline conditions and following infection with *K. pneumoniae*. Towards this goal we used one-dimensional gels and 2D-DIGE to compare the BAL proteomes of C57BL/6 mice and SP-A-/- mice on the same genetic background under baseline conditions. We then characterized changes in the BAL proteome at 4 and 24 hr after intratracheal infection with *K. pneumoniae *and compared the responses between the two strains of mice in order to gain insight into molecules and pathways involved in the presence and absence of SP-A.

### 1-D gel analysis of BAL proteins from *K. pneumoniae*-infected WT and SP-A-/- mice

**Figure 1:**
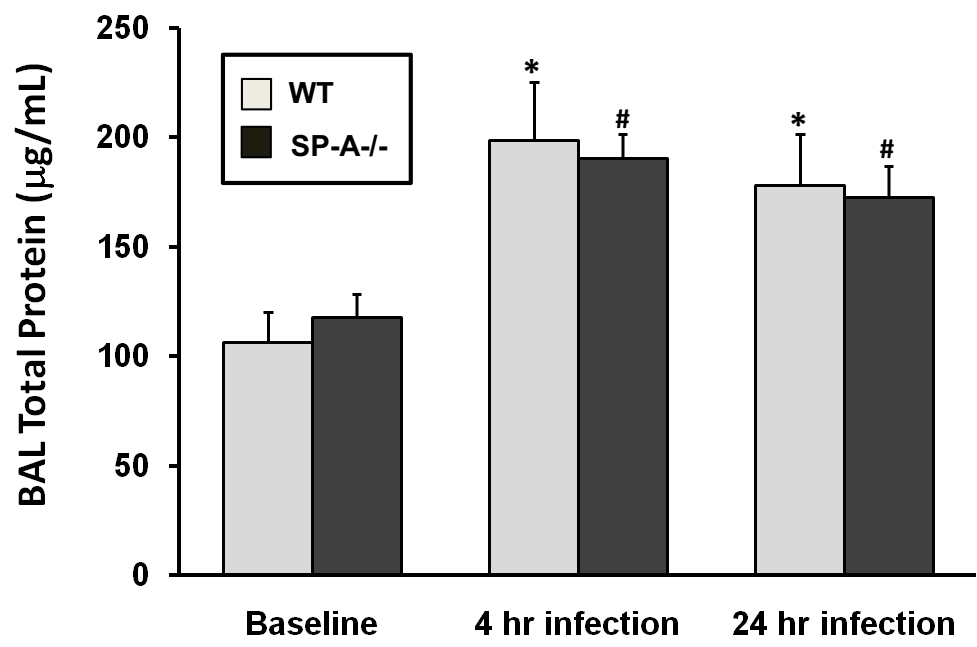
**BAL total protein concentration**. The total protein concentration of cell-free BAL fluid was determined. The total amount of fluid recovered during BAL (~90% of instilled volume) did not differ significantly among groups. The histogram depicts mean protein concentration values (n = 4/group) and standard deviations are indicated by error bars. Values of WT mice that differ significantly (p < 0.05) from baseline by t-tests are indicated by an asterisk (*) and values of SP-A-/- mice that differ from baseline values are indicated with a pound sign (#).

Although there were no significant differences among groups in the amount of fluid recovered by BAL, significant (p < 0.05) increases in total protein concentration were observed in infected (4 hr and 24 hr) WT and SP-A-/- mice when compared with the corresponding untreated mice (Figure 1). However, there were no significant differences in total BAL protein between WT and SP-A-/- mice under any of the experimental conditions. It should be noted that this increase, based on the results that will be described subsequently, does not appear to be due to increased permeability and an influx of serum proteins into the alveolar space. This is evident from the fact that abundant serum proteins (i.e. albumin, ceruloplasmin, haptoglobin, prothrombin, etc.) are not increasing in proportion to the changes in total protein concentration.

One-dimensional gel analysis (data not shown) revealed a band of potential interest at ~14 kDa which appeared with higher intensity in SP-A-/- control mice compared to WT control mice. However, as infection progressed, the intensity of the 14 kDa band decreased in the SP-A-/- mice and increased in the WT mice, indicating that this 14 kDa protein may play a role in host defense. MALDI-ToF/ToF mass spectrometry analysis of the 14 kDa protein band identified it as lysozyme, a protein known to have antibacterial properties [18,30].

### 2D-DIGE analysis

**Figure 2:**
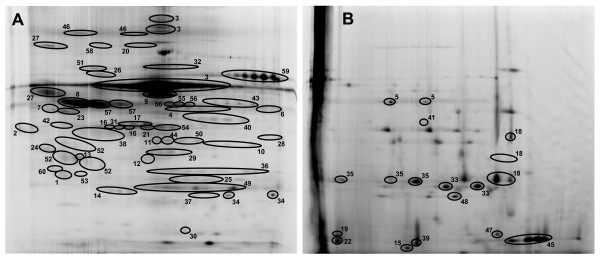
**Reference gels**. The reference gels for two-dimensional separations in pH 4-7 gels (A) and pH 7-11 gels (B) are shown. Protein spots that have been identified by MALDI-ToF/ToF are circled and numbered and all proteins are named in Table 1. Note that in cases where multiple isoforms are circled, the identity of all isoforms has been confirmed by MALDI-ToF/ToF.

To provide the broadest proteomic coverage, two different pH gradients were used in the first dimension gels. One employed a pH 4-7 gradient and the other used a pH 7-11 gradient. We chose the pH 7-11 gradient because the isoelectric point of lysozyme is ~pH 11 and the lysozyme content was seen to change between SP-A-/- and WT mice in the presence and absence of infection in the 1-D analysis. This gradient allowed us to resolve some of the very basic proteins, such as lysozyme, that would not typically be resolved in pH 3-10 gels. The pH 4-7 gradient gel was used because many proteins in this pH range are somewhat compressed when a pH 3-10 gradient is used. However, it is possible that there may have been some proteins in very acidic and more basic ranges that were not resolved. A 10-14% polyacrylamide gradient was used for the second dimension separation to allow a better resolution of lower molecular weight proteins, although the resolution of some proteins with higher molecular weights was slightly compromised. The 2D-DIGE analysis involved 715 protein spots of which 279 spots constituting 60 proteins were visualized in all samples and identified by MALDI-ToF/ToF (Table 1). The identified proteins accounted for 93% of the detectable protein on the pH 4-7 gels (Figure 2A) and 39% on the pH 7-11 gels (Figure 2B).

**Table 1:** List of identified proteins

**Gel No**.	Protein	**Accession No**.	Functional Group	IPG
1	14-3-3-Zeta	P63101	DEF, RED, PMM	4-7
2	Adipsin (Complement factor D)	P03953	DEF, PMM	4-7
3	Albumin	P07724	RED	4-7
4	Aldehyde dehydrogenase AHD-M1	P47738	RED	4-7
5	Aldehyde dehydrogenase II	P24549	RED	7-11
6	Aldehyde dehydrogenase, Dimeric NADP-preferring (EC 1.2.1.5) (ALDH class 3)	P47739	RED	4-7
7	Alpha-1-antitrypsin 1-1 precursor (Serine protease inhibitor 1-1)	P07758	DEF, PMM	4-7
8	Alpha-1-antitrypsin 1-6 precursor (Serine protease inhibitor 1-6) (Alpha-1 protease inhibitor)	P81105	DEF, PMM	4-7
9	Alpha-fetoprotein	P02772		4-7
10	Annexin A1 (Annexin I) (Lipocortin I) (Calpactin II) (Chromobindin-9) (P35)	P10107	DEF	4-7
11	Annexin A3	O35639		4-7
12	Annexin A4	Q3UCL0		4-7
13	Annexin A5	P48036	DEF	4-7
14	Apolipoprotein A-1	Q58EV2	DEF, RED	4-7
15	Beta-2-microglobulin	Q91XJ8	DEF	7-11
16	Beta-actin	P60709		4-7
17	Beta-actin (putative, AA 27-375) (alpha-actin)	Q61275		4-7
18	Carbonyl reductase 2	P08074	RED	7-11
19	Cell specific 10K protein (uteroglobin - mouse) (Clara cell 10K protein) (CC10) (CC16)	Q06318	DEF, RED	7-11
20	Ceruloplasmin isoforms	Q61147	RED	4-7
21	Chain A, The Crystal structure of novel mammalian lectin Ym1-suggests a saccharide binding site	O35744	DEF	4-7
22	Chain B, Chimeric human mouse carbonmonoxyhemoglobin (Human zeta, Mouse beta 2)	P02088		7-11
23	Chia protein	Q91XA9	DEF	4-7
24	Coiled-coil domain containing 122	Q8BVN0		4-7
25	Complement component 3	Q80XP1	DEF	4-7
26	Complement component C5	P06684	DEF	4-7
27	Contrapsin (Serine protease inhibitor A3K)	P07759	PMM	4-7
28	Creatine kinase M-type (EC.2.7.3.2) (Creatine kinase M chain) (M-CK)	P07310		4-7
29	Cytosolic malate dehydrogenase	P14152	RED	4-7
30	(Similar to) Ferritin light chain 1 (Ferritin L, subunit 1)	P29391	DEF, RED	4-7
31	Gamma-actin	P63260		4-7
32	Gelsolin precursor (Actin-depolymerizing factor) (ADF) (Brevin)	P13020	DEF, RED, PMM	4-7
33	Glutathione S-transferase, alpha 3	P30115	DEF, RED	7-11
34	Glutathione S-transferase, alpha 4	P24472	DEF, RED	4-7
35	Glutathione S-transferase, mu 1	P10649	DEF, RED	7-11
36	Glutathione S-transferase, omega 1	O09131	DEF, RED	4-7
37	(Similar to) Glutathione S-transferase, Ya chain (GST class-alpha) (Ya1)	P13745	DEF, RED	4-7
38	Haptoglobin	Q60574	DEF, RED	4-7
39	Hemoglobin subunit alpha (Hemoglobin alpha chain)(Alpha-globin)	P01942		7-11
40	Isocitrate dehydrogenase [NADP] cytoplasmic (EC 1.1.1.42) (Cytosolic NADP-Isocitr)	O88844	RED	4-7
41	Keratin complex 1, acidic, gene 10	P02535		7-11
42	(Similar to) Keratin, type I cytoskeletal 10 (Cytokeratin-10) (CK-10) (Keratin-10)	A2A513		4-7
43	Kpnb1 protein b	Q99KM9		4-7
44	Lactate dehydrogenase 2, B chain	P16125	DEF, RED	4-7
45	Lysozyme 2	P08905	DEF, RED	7-11
46	Murinoglobulin-1 precursor (MuG1)	P28665	DEF, PMM	4-7
47	Myosin heavy chain IIB	Q9JHR4		7-11
48	Peroxiredoxin 1	P35700	RED	7-11
49	Peroxiredoxin 6	Q6GT24	DEF, RED	4-7
50	Pregnancy zone protein	Q61838	DEF, PMM	4-7
51	Prothrombin precursor (Ec 3.4.21.5) (Coagulation factor II)	P19221	DEF, PMM	4-7
52	Pulmonary surfactant associated protein A precursor (SP-A) (PSP-A) (PSAP)	P35242	DEF, RED	4-7
53	Rho GDP dissociation inhibitor (GDI) alpha	Q99PT1		4-7
54	SEC14-like 3	Q5SQ27	PMM	4-7
55	Selenium binding protein 1	P17563	DEF, RED	4-7
56	Selenium binding protein 2	Q63836	DEF, RED	4-7
57	Serine (or cysteine) proteinase inhibitor, clade A, member 1e	Q00898	PMM	4-7
58	Toll-like receptor 13 precursor	Q6R5N8	DEF	4-7
59	Transferrin	Q921I1	DEF, RED, PMM	4-7
60	Tyrosine-3-monooxygenase/tryptophan 5-monooxynase activation protein, Epsilon polypeptide	Q8BPH1	PMM	4-7

Proteins identified by 2D-DIGE. Gel numbers (see Fig. 2), protein names, SwissProt accession numbers, functional groups and pH gradients (IPG) of the gel where the proteins were identified are listed. Abbreviations for functional groups: DEF, host defense; PMM, protein modification and metabolism; RED, redox regulation.

Whole BAL supernatant samples (without serum protein depletion) were used for both pH ranges. Depletion of serum proteins results in substantial reductions in the amount of protein available and would have precluded us from performing the two first-dimensional separations with different pH ranges. Below we present and compare changes in the BAL proteome of WT and SP-A-/- mice. We studied: 1) the levels of expression of specific proteins under baseline conditions, 2) levels of expression at each of two time points after infection, and 3) comparisons between the two mouse strains, namely the patterns of changes in expression during the course of infection.

#### 1) Baseline conditions

This is the first time that the BAL proteome of SP-A-/- mice has been compared to their WT counterpart under baseline conditions. In this state the differences between the two strains are rather striking, although the responses to infection that will be described subsequently show many similarities. Of the 60 proteins studied (59 when SP-A is excluded), there were decreases in levels of expression of more than 40 proteins, 24 of these decreasing by more than 25%. Increases were seen in 19 proteins, 7 of these increasing by more than 25%. Out of all of these changes, there were significant differences in the levels of expression of 14 proteins, 11 of these significant changes being decreases (Table 2).

**Table 2:** Changes in protein expression between wild-type and SP-A-/- mice for control, 4 hr post infection and 24 hr post infection: percent changes with significance for all identified proteins corresponding to reference gels in Fig. 2

**Gel No**.	Protein	Functional Group	% Δ WT to SP-A-/- Baseline	% Δ WT to SP-A-/- 4 hr	% Δ WT to SP-A-/- 24 hr
1	14-3-3-Zeta	DEF, RED, PMM	-34.97	16.24	-10.69
2	Adipsin (Complement factor D)	DEF, PMM	-63.51	5.95	4.76
3	Albumin	RED	4.82	1.56	12.73
4	Aldehyde dehydrogenase AHD-M1	RED	-82.62	37.46	29.21
5	Aldehyde dehydrogenase II	RED	**135.85**	14.63	31.75
6	Aldehyde dehydrogenase, Dimeric NADP-preferring (EC 1.2.1.5) (ALDH class 3)	RED	58.04	**40.10**	38.77
7	Alpha-1-antitrypsin 1-1 precursor (Serine protease inhibitor 1-1)	DEF, PMM	-30.98	15.12	16.63
8	Alpha-1-antitrypsin 1-6 precursor (Serine protease inhibitor 1-6) (Alpha-1 protease inhibitor)	DEF, PMM	-12.72	-9.38	-12.84
9	Alpha-fetoprotein		-16.1	24.43	-11.61
10	Annexin A1 (Annexin I) (Lipocortin I) (Calpactin II) (Chromobindin-9) (P35)	DEF	-201.08	72.45	4.89
11	Annexin A3		21.76	-7.73	1.22
12	Annexin A4		13.78	14.08	3.38
13	Annexin A5	DEF	**-381.16**	-66.73	**-130.01**
14	Apolipoprotein A-1	DEF, RED	-23.3	**-89.53**	93.80
15	Beta-2-microglobulin	DEF	68.18	**-59.79**	**-55.69**
16	Beta-actin		-14.27	**32.95**	13.93
17	Beta-actin (putative, AA 27-375) (alpha-actin)		4.84	**52.39**	9.19
18	Carbonyl reductase 2	RED	**-184.18**	29.93	0.81
19	Cell specific 10K protein (uteroglobin - mouse) (Clara cell 10K protein) (CC10) (CC16)	DEF, RED	-73.76	12.90	64.31
20	Ceruloplasmin isoforms	RED	22.02	-9.13	-19.71
21	Chain A, Crystal structure of novel mammalian lectin Ym1-suggests a saccharide binding site	DEF	-2.98	**28.89**	13.35
22	Chain B, Chimeric human mouse carbonmonoxyhemoglobin (Human zeta, Mouse beta 2)		-5.61	8.67	33.51
23	Chia protein	DEF	5.35	**31.40**	17.25
24	Coiled-coil domain containing 122		**-183.85**	21.31	-6.43
25	Complement component 3	DEF	-12.5	**40.66**	8.39
26	Complement component C5	DEF	-22.76	21.75	5.19
27	Contrapsin (Serine protease inhibitor A3K)	PMM	7.77	-8.52	-8.74
28	Creatine kinase M-type (EC.2.7.3.2) (Creatine kinase M chain) (M-CK)		-57.22	28.34	-22.83
29	Cytosolic malate dehydrogenase	RED	-3.05	7.03	1.31
30	(Similar to) Ferritin light chain 1 (Ferritin L, subunit 1)	DEF, RED	**-87.39**	-14.84	-3.35
31	Gamma-actin		-20.73	31.85	20.75
32	Gelsolin precursor (Actin-depolymerizing factor) (ADF) (Brevin)	DEF, RED, PMM	30.02	-3.09	-2.90
33	Glutathione S-transferase, alpha 3	DEF, RED	**-193.11**	21.05	19.07
34	Glutathione S-transferase, alpha 4	DEF, RED	-339.42	64.33	38.47
35	Glutathione S-transferase, mu 1	DEF, RED	**-101.12**	45.92	35.09
36	Glutathione S-transferase, omega 1	DEF, RED	**-132.89**	**48.91**	15.84
37	(Similar to) Glutathione S-transferase, Ya chain (GST class-alpha) (Ya1)	DEF, RED	-318.76	56.63	44.05
38	Haptoglobin	DEF, RED	-14.69	16.92	2.41
39	Hemoglobin subunit alpha (Hemoglobin alpha chain)(Alpha-globin)		-6.6	-32.60	132.59
40	Isocitrate dehydrogenase [NADP] cytoplasmic (EC 1.1.1.42) (Cytosolic NADP-Isocitr)	RED	-21.59	17.31	1.14
41	Keratin complex 1, acidic, gene 10		16.11	20.63	-30.55
42	(Similar to) Keratin, type I cytoskeletal 10 (Cytokeratin-10) (CK-10) (Keratin-10)		**-43.31**	26.68	-5.45
43	Kpnb1 protein b		-55.63	**53.13**	34.52
44	Lactate dehydrogenase 2, B chain	DEF, RED	**28.46**	-4.34	-3.26
45	Lysozyme 2	DEF, RED	129.29	**-63.56**	**-133.43**
46	Murinoglobulin-1 precursor (MuG1)	DEF, PMM	-14.21	**-16.51**	-6.93
47	Myosin heavy chain IIB		-21.97	6.81	-15.67
48	Peroxiredoxin 1	RED	**-83.18**	24.93	11.30
49	Peroxiredoxin 6	DEF, RED	-54.85	**56.63**	**42.36**
50	Pregnancy zone protein	DEF, PMM	-1.56	2.46	-7.38
51	Prothrombin precursor (Ec 3.4.21.5) (Coagulation factor II)	DEF, PMM	-59.43	6.39	5.13
52	Pulmonary surfactant associated protein A precursor (SP-A) (PSP-A) (PSAP)	DEF, RED	**ND**	**ND**	**ND**
53	Rho GDP dissociation inhibitor (GDI) alpha		**-115.4**	-22.62	-42.11
54	SEC14-like 3	PMM	-33.59	30.46	9.92
55	Selenium binding protein 1	DEF, RED	9.26	-4.10	-3.32
56	Selenium binding protein 2	DEF, RED	**27.17**	-2.40	-3.86
57	Serine (or cysteine) proteinase inhibitor, clade A, member 1e	PMM	7.7	**-18.15**	-11.72
58	Toll-like receptor 13 precursor	DEF	18.41	**-17.04**	-4.57
59	Transferrin	DEF, RED, PMM	8.65	**-17.12**	-0.37
60	Tyrosine-3-monooxygenase/tryptophan 5-monooxynase activation protein, Epsilon polypeptide	PMM	**-183.11**	18.41	-7.70

Changes (% Δ) in BAL protein expression from wild-type (WT) to SP-A-/- mice are shown for untreated (baseline) animals, 4 hours post-infection and 24 hours post-infection. Gel numbers (see Fig. 2), protein names, functional groups and percent change (% Δ) are listed. Abbreviations for functional groups: DEF, host defense; PMM, protein modification and metabolism; RED, redox regulation. Bolded numbers indicate changes that were significant (p < 0.05 by t-test). Values for SP-A, which is absent in SP-A-/- mice, are listed as not determined (ND).

In order to help elucidate the potential functional significance of these changes we assigned the identified proteins to several broad functional classes based on reviewing the published studies reporting the functions of these proteins (see Table 1). The classes were: a) host defense proteins (DEF); b) proteins involved in regulating redox balance (RED); and c) proteins involved in protein metabolism and modification (PMM). It should be noted that some proteins are in more than one functional group. This classification scheme, which we have used in other studies [13,29] was more inclusive than relying solely on the biological function classification provided by PANTHER and similar gene ontology databases. Other authors have focused on groups of proteins involved in host defense and oxidative stress in studies of the BAL proteome in response to exogenous glucocorticoid treatment or stress [26,27]. We analyzed the changes in these functional groups to determine whether we could gain insight into the basis for: a) the phenotype of the SP-A-/- mice, specifically their greater susceptibility to infection and increased mortality due to infection [12]; and b) the tendency of SP-A-/- mice (compared to WT mice) to experience an aggravated response to oxidative stress resulting from an acute ozone exposure that we have described previously [13,31].

More than half of the identified proteins (n = 31, excluding SP-A) were designated DEF proteins (Table 1). Roughly 2/3 of these proteins (n = 22) were expressed at lower levels in SP-A-/- mice than in WT animals, and 14 of these proteins showed at least a 25% reduction from WT levels. Of the 7 DEF proteins that differed significantly between strains, 5 of these were at lower levels in the SP-A-/- mice and 2 were at higher levels, as was lysozyme (although its increase did not achieve statistical significance). SP-A was not included in these comparisons because of its absence in the SP-A-/- mice.

Proteins in the RED group exhibited a similar pattern. This group consisted of 26 proteins and 16 of them were at decreased levels in the SP-A-/- mice, with 12 of the 16 proteins showing at least a 25% reduction. Six of the 9 RED proteins differing significantly between strains were decreased in SP-A-/- mice. A similar trend was also noted in PMM proteins (n = 13) where 9 of the proteins showed reduced levels of expression in the SP-A-/- mice.

Notable among the decreased proteins in the SP-A-/- mice were annexins A1 and A5, apolipoprotein A-1, several complement components, several glutathione transferases, and peroxiredoxin 6. These proteins have all been attributed protective roles against infection, inflammation, and reactive oxidants, and their decreased levels in the SP-A-/- mice, together with the absence of SP-A, suggest that these mice could be vulnerable to a wide range of insults. SP-A-/- mice exhibit increased susceptibility to infection, reduced survival, reduced macrophage activation, inability to combat infection, and an exaggerated response to oxidative stress [9,10,12,31-33]. However, there are also some indications of possible compensatory measures to counter the host defense deficits resulting from the lack of SP-A. Lysozyme and β-2-microglobulin, both of which serve in pathogen defense in mice [18,19] were elevated in the SP-A-/- mice under baseline conditions.

One could argue, based on the predominance of proteins exhibiting reduced levels of expression in the SP-A-/- mice, that there was a non-specific reduction in the production and secretion of BAL proteins in this group. However, this possibility is refuted by the fact that the protein content of BAL from both strains does not differ significantly (Figure 1) and there are a number of proteins whose levels of expression are higher in SP-A-/- mice. However, the fact that many DEF and RED proteins are reduced in the SP-A-/- mice under baseline conditions is consistent with prior reports that the alveolar macrophages of SP-A-/- mice are dysfunctional or hypoactive [12]. This finding leads us to speculate that the absence of SP-A results in reduced levels of regulatory molecules produced by these macrophages that may be responsible for these lower levels of expression of DEF and RED proteins.

#### 2) Responses to K. pneumoniae infection

We compared the proteins expressed by WT and SP-A-/- mice at each of the two time points following infection (Table 2), as well as the pattern of changes in protein expression between time points in each strain (Tables 3 and 4).

**Table 3:** List of proteins with similar changes in both strains of mice between 4 hours and 24 hours post infection

**Gel No**.	Protein	**Accession No**.	% Δ WT	% Δ SP-A-/-
1	14-3-3-Zeta	P63101	35.39	5.23
2	Adipsin (Complement factor D)	P03953	-3.11	-4.28
4	Aldehyde dehydrogenase AHD-M1	P47738	-0.68	-7.11
5	Aldehyde dehydrogenase II	P24549	-25.89	-9.53
6	Aldehyde dehydrogenase, Dimeric NADP-preferring (EC 1.2.1.5) (ALDH class 3)	P47739	-4.45	-5.45
7	Alpha-1-antitrypsin 1-1 precursor (Serine protease inhibitor 1-1)	P07758	11.73	13.19
8	Alpha-1-antitrypsin 1-6 precursor (Serine protease inhibitor 1-6) (Alpha-1 protease inhibitor)	P81105	-1.97	-5.19
9	Alpha-fetoprotein	P02772	**58.71**	14.27
10	Annexin A1 (Annexin I) (Lipocortin I) (Calpactin II) (Chromobindin-9) (P35)	P10107	**104.30**	24.23
11	Annexin A3	O35639	-11.62	-2.36
15	Beta-2-microglobulin	Q91XJ8	-11.91	-9.04
17	Beta-actin (putative, AA 27-375) (alpha-actin)	Q61275	**98.26**	42.05
18	Carbonyl reductase 2	P08074	-14.63	**-47.75**
19	Cell specific 10K protein (uteroglobin - mouse) (Clara cell 10K protein) (CC10) (CC16)	Q06318	189.97	322.02
20	Ceruloplasmin isoforms	Q61147	9.88	0.00
21	Chain A, The Crystal structure of novel mammalian lectin Ym1-suggests a saccharide binding site	O35744	**29.60**	13.98
22	Chain B, Chimeric human mouse carbonmonoxyhemoglobin (Human zeta, Mouse beta 2)	P02088	**42.79**	**75.43**
23	Chia protein	Q91XA9	**27.11**	13.42
24	Coiled-coil domain containing 122	Q8BVN0	**55.69**	20.59
27	Contrapsin (Serine protease inhibitor A3K)	P07759	8.67	8.44
28	Creatine kinase M-type (EC.2.7.3.2) (Creatine kinase M chain) (M-CK)	P07310	**103.29**	28.96
30	(Similar to) Ferritin light chain 1 (Ferritin L, subunit 1)	P29391	-32.41	-19.16
32	Gelsolin precursor (Actin-depolymerizing factor) (ADF) (Brevin)	P13020	**-11.18**	-10.99
33	Glutathione S-transferase, alpha 3	P30115	33.97	9.01
34	Glutathione S-transferase, alpha 4	P24472	28.76	**8.61**
35	Glutathione S-transferase, mu 1	P10649	-3.93	-12.25
36	Glutathione S-transferase, omega 1	O09131	32.86	3.36
37	(Similar to) Glutathione S-transferase, Ya chain (GST class-alpha) (Ya1)	P13745	17.64	8.19
39	Hemoglobin subunit alpha (Hemoglobin alpha chain) (Alpha-globin)	P01942	47.17	353.87
41	Keratin complex 1, acidic, gene 10	P02535	**125.91**	43.46
43	Kpnb1 protein b	Q99KM9	-0.48	-14.37
44	Lactate dehydrogenase 2, B chain	P16125	-9.56	-8.43
46	Murinoglobulin-1 presursor (MuG1)	P28665	-14.22	-4.83
47	Myosin heavy chain IIB	Q9JHR4	**59.33**	28.97
48	Peroxiredoxin 1	P35700	20.27	7.45
51	Prothrombin precursor (Ec 3.4.21.5) (Coagulation factor II)	P19221	8.81	7.52
52	Pulmonary surfactant associated protein A precursor (SP-A) (PSP-A) (PSAP)	P35242	37.49	ND
53	Rho GDP dissociation inhibitor (GDI) alpha	Q99PT1	48.73	28.33
55	Selenium binding protein 1	P17563	-1.65	-0.89
56	Selenium binding protein 2	Q63836	-2.42	-3.88
57	Serine (or cysteine) proteinase inhibitor, clade A, member 1e	Q00898	-9.83	-3.86
60	Tyrosine-3-monooxygenase/tryptophan 5-monooxynase activation protein, Epsilon polypeptide	Q8BPH1	33.02	4.31

Changes (% Δ) in BAL protein expression that were similar for wild-type (WT) and SP-A-/- mice between 4 and 24 hours post infection are shown. Gel numbers (see Fig. 2), protein names, SwissProt accession numbers and percent change (% Δ) for both WT and SP-A-/- are listed. Bolded numbers indicate changes that were significant (p < 0.05 by t-test) between 4 and 24 hours for a given group. Values for SP-A, which is absent in SP-A-/- mice are listed as not detectable (ND) in SP-A-/- mice.

**Table 4:** List of proteins with different changes for strains of mice between 4 hours and 24 hours post infection

**Gel No**.	Protein	**Accession No**.	% Δ WT	% Δ SP-A-/-
3	Albumin	P07724	-3.61	7.12
12	Annexin A4	Q3UCL0	3.05	-7.09
13	Annexin A5	P48036	**23.19**	-11.99
14	Apolipoprotein A-1	Q58EV2	-166.30	37.97
16	Beta-actin	P60709	11.33	-4.82
25	Complement component 3	Q80XP1	5.47	**-23.04**
26	Complement component C5	P06684	12.19	-3.16
29	Cytosolic malate dehydrogenase	P14152	2.02	-3.55
31	Gamma-actin	P63260	0.67	-8.47
38	Haptoglobin	Q60574	1.80	-12.16
40	Isocitrate dehydrogenase [NADP] cytoplasmic (EC 1.1.1.42) (Cytosolic NADP-Isocitr)	O88844	**10.70**	-4.77
42	(Similar to) Keratin, type I cytoskeletal 10 (Cytokeratin-10) (CK-10) (Keratin-10)	A2A513	24.80	-7.04
45	Lysozyme 2	P08905	4.36	-36.75
49	Peroxiredoxin 6	Q6GT24	4.30	-5.42
50	Pregnancy zone protein	Q61838	5.37	-4.41
54	SEC14-like 3	Q5SQ27	8.92	-8.97
58	Toll-like receptor 13 precursor	Q6R5N8	-4.87	6.73
59	Transferrin	Q921I1	-12.29	3.92

Changes (% Δ) in BAL protein expression that were different for wild-type (WT) and SP-A-/- mice between 4 and 24 hours post infection are shown. Gel numbers (see Fig. 2), protein names, SwissProt accession numbers and percent change (% Δ) for both WT and SP-A-/- are listed. Bolded numbers indicate changes that were significant (p < 0.05 by t-test) between 4 and 24 hours for a given group.

##### a) Strain differences at 4 hr after infection

As compared to uninfected animals, we observed a very different picture when we compared strains following infection with *K. pneumoniae*. Four hr after infection the levels of more than two-thirds (n = 40) of the identified BAL proteins in SP-A-/- mice were increased as compared to WT mice. The proteins found to be increased in SP-A-/- mice after infection (Table 2) included many of the proteins that had been found to be decreased under baseline conditions in uninfected SP-A-/- mice as compared to WT mice. Our working hypotheses to explain these differences are: a) that expression of these proteins is stimulated by the induction of infection, probably as a consequence of increased levels of regulatory molecules such as cytokines; b) in order to compensate for the lower baseline levels of many proteins in SP-A-/- mice, expression of these proteins is strongly stimulated or overcompensated with infection in SP-A-/- mice; and c) the absence, or deficit, of some (as yet unidentified) regulatory molecules in the SP-A-/- mice (as postulated above) results in a poorly regulated, overexuberant response to infection so that levels of expression after infection often exceed those seen in WT mice. It is also of interest to note that several of the proteins (13 proteins out of 59) that were present in increased amounts in the uninfected SP-A-/- mice respond to infection by reducing their levels below those seen in the corresponding infected WT mice, resulting in a regulatory pattern that is almost the inverse of that described above for most of the proteins. These proteins decreased in infected SP-A-/- mice include both lysozyme and β_2_-microglobulin. A number of the proteins mentioned above are included in the categories of positive and negative acute phase proteins and the patterns of expression we describe are consistent with this classification.

Together, the observations made and the hypotheses stated earlier indicate that in the absence of SP-A there is a loss of regulatory control to appropriately modulate expression of DEF proteins in response to infection. Consistent with this postulate, a recent study with SP-A-/- mice demonstrated that in response to low level intrapharyngeal LPS treatment (0.5 ng), significantly higher levels of MIP-2 were observed compared to the untreated control [34]. However, in this study similarly treated WT mice did not have increases in MIP-2 at low LPS doses, but required a higher LPS concentration to exhibit a similar increase [34]. Interestingly, a recent study in which LPS was administered into a lobe of healthy volunteers' lung found significant decreases in levels of SP-A [28]. Unfortunately, there was little overlap in the sets of identified proteins between our studies and therefore little basis for comparison.

When the DEF and RED proteins are individually examined as subgroups of the identified proteins, the changes are similar to those noted for all proteins. In both of these groups of proteins, more proteins show increased levels of expression in infected SP-A-/- mice vs infected WT mice (18 DEF proteins; n = 17 RED proteins), a situation that is almost the exact opposite of what was seen in untreated mice and consistent with the loss of regulatory control we propose in the SP-A-/- mice.

It is not yet known what causes these rapid changes. They may result from the presence of bacteria, the influx of immune cells to combat the bacteria, or the release of mediator(s) from immune cells or epithelium to deal with the insult. We speculate that the rapidity of the response (within 4 hr) is due to the release of stored mediators, such as chemokines, rather than due to the synthesis and secretion of new protein. As stated earlier, because many of the identified proteins were at lower levels in the untreated SP-A-/- mice, the post-infection response may be an effort to restore these proteins to levels that can reduce the threat posed by the instilled bacteria. Two proteins that may play important roles in host defense and have been shown to contribute specifically to host defense against *K. pneumoniae*, lysozyme and β-2-microglobulin, were of particular interest [18,19]. These proteins exhibited higher levels in the untreated SP-A-/- mice, perhaps in an effort to bolster the compromised host defense status of these mice, but following infection their levels in the SP-A-/- mice dropped to below those seen in WT mice. A consistent finding was observed for lysozyme by 1-D gel analysis (data not shown). Whether this reduction in the levels of these proteins was a consequence of their involvement and elimination in the course of defense processes against the instilled bacteria or a reduction in their synthesis remains to be determined.

##### b) Strain differences at 24 hr after infection

Extending the analysis to mice infected 24 hr earlier we gained some additional insight into the response pattern. Three general trends were seen. 1) In one set of proteins (n = 11) there was little change between 4 and 24 hr. 2) In another set (n = 36), at the 24 hr point there were proteins in which the levels of expression tended to revert back (⋀- or ⋁-shaped response pattern) toward the levels seen under baseline conditions, suggesting a peak response at 4 hr (or at least earlier than 24 hr). 3) In a third set (n = 5), levels of some proteins at 24 hr were continuing to either increase or decrease from levels seen at 4 hr, suggesting slower and/or more sustained responses to infection. With respect to all identified proteins, the ratio of increased to decreased proteins (34/25 = 1.36) at 24 hr is lower than at 4 hr (40/19 = 2.1), although the increases continue to predominate. However, most of this change is due to the PMM group, which has reverted at the 24 hr point post-infection to having more than twice as many proteins (9 of 13) at reduced levels in the SP-A-/- mice, as was the case in control (baseline) SP-A-/- mice. The overall numbers of increased and decreased expression levels of DEF and RED proteins at the 24 hour point are quite similar to those seen at 4 hr after infection. However, there were a few notable exceptions. For example, apolipoprotein A-1, which is known to exhibit anti-inflammatory activity [35], was lower in SP-A-/- mice than WT mice both at baseline (-23.3%) and after 4 hr of infection (-89.53%), but by the 24 hour time point its levels were markedly higher in the SP-A-/- mice (93.8%), indicating a delayed response in the SP-A-/- mice.

##### c) Potential pathways affected by changes

**Figure 3:**
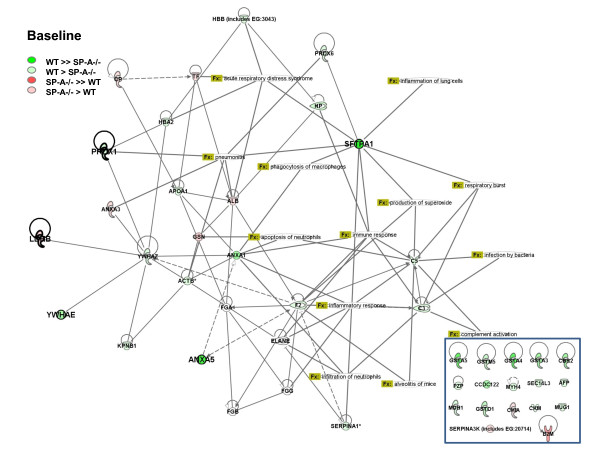
**Network analysis of BAL proteome changes under baseline conditions**. Changes in levels of expression between WT and SP-A-/- mice under baseline conditions and at 4 hr after infection (Figure 4) were analyzed using the Ingenuity Pathway program focusing on pathways related to lung disease (shown as Function (Fx) in olive green squares). Proteins undergoing significant changes are indicated in bold. Changing proteins that were not included in the pathway are shown in the inset in the lower right corner. The relationship between symbol color and relative levels of expression is shown in the upper left corner. Unshaded proteins in the pathway were not identified in our gels. The proteins included in the analysis and the Ingenuity abbreviation are listed below (see also Table 1): Cytosolic malate dehydrogenase: MDH1; Ferritin light chain 1: FTL; Gamma-actin: ACTG1; Gelsolin: GSN; Glutathione S-transferase, alpha 3: GSTA3; Glutathione S-transferase, alpha 4: GSTA4; Glutathione S-transferase, mu 1: GSTM5; Glutathione S-transferase, omega 1: GSTO1; Glutathione S-transferase, Ya chain (GST class-alpha): GSTA5; Haptoglobin: HP; Hemoglobin subunit alpha: HBA2; Isocitrate dehydrogenase: IDH1; Keratin complex 1, acidic, gene 10: KRT10; Kpnb1 protein b: KPNB1; Lactate dehydrogenase 2, B chain: LDHB; Lysozyme 2:; LYZ; Murinoglobulin-1 precursor: MUG1; Myosin heavy chain IIB: MYH4; Peroxiredoxin 1: PRDX1; Peroxiredoxin 6: PRDX6; Pregnancy zone protein: PZP; Prothrombin precursor: F2; Pulmonary surfactant associated protein A precursor: SFTPA1; Rho GDP dissociation inhibitor (GDI) alpha: ARHGDIA; SEC14-like 3: SEC14L3; Selenium binding protein 1: SELENBP1; Toll-like receptor 13 precursor: TLR13; Transferrin: TF; Tyrosine-3-monooxygenase/tryptophan 5-monooxygenase activation protein, Epsilon polypeptide: YWHAE

**Figure 4:**
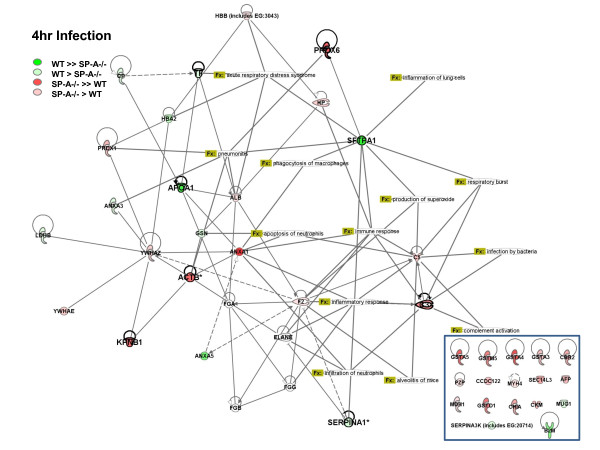
**Network analysis of BAL proteome changes at 4 hours after infection**. Changes in levels of expression between WT and SP-A-/- mice under baseline conditions (Figure 3) and at 4 hr after infection (Figure 4) were analyzed using the Ingenuity Pathway program. A description of the figure and the abbreviations used are the same as those in the legend for Figure 3.

We also used the Ingenuity Systems Pathways Analysis program to better understand the functional implications of the absence of SP-A, both under baseline conditions and after infection with *K. pneumoniae*. These analyses are depicted in Figure 3 and provide a graphic demonstration of the trends we described from our statistical analysis. Roughly half of the identified proteins were included in a network with components of this network having roles in a variety of biological processes that are relevant to our experimental model. These include infection by bacteria, inflammatory response, complement activation, phagocytosis of macrophages, and others. Proteins that were not linked in the network are shown in the inset. In the diagram reflecting baseline conditions (Fig. 3A) the majority of proteins are colored green, reflecting levels of expression in the SP-A-/- mice below those seen in WT mice, as we described earlier. A very different picture is seen when the same comparison is made at 4 hr after infection (Fig. 4). At this time point most of the proteins are red, representing the fact that expression is higher in SP-A-/- mice than in WT mice and indicating that in the SP-A-/- mice the synthesis and/or secretion of these proteins is rapidly enhanced by infection.

#### 3) Comparison of changes that occur in each strain between 4 and 24 hr after infection

A comparison of the infection-induced response between 4 and 24 hr in WT and SP-A-/- mice, was made by calculating changes in the levels of specific proteins between 4 and 24 hr of infection in each strain, and comparing the WT and SP-A-/- mice. This analysis showed the following. The first difference of note is that changes between 4 and 24 hr in 13 of the 59 identified proteins were statistically significant in the WT mice, 12 of the 13 being significant increases (Tables 3 and 4). By contrast, there were only 4 significant changes between 4 and 24 hr in the SP-A-/- mice. However, in general the response patterns were quite similar in both strains with 41 proteins (excluding SP-A) showing either increases or decreases in both strains (Table 3) and 18 more that showed increases in one strain and decreases in the other (Table 4). The similarities and differences between strains in the responses are described below in more detail.

##### a) Similar responses in both strains

Following infection of mice with *K. pneumoniae *there were a number of proteins that underwent similar changes between 4 and 24 hr after infection, typically increases, in both strains of mice (Table 3). The first group of note includes beta-actin, myosin IIB (a non-muscle myosin), creatine kinase M, and Rho GDP dissociation inhibitor-alpha. All of these are proteins that play a role in phagocytosis [36] and are increased in both strains. These increases could be related to the presence of increased numbers of phagocytic cells to combat the infection. In all cases the amount of increase was greater in the WT mice and for most of the proteins the changes attained statistical significance only in WT mice.

Also notable were the increases in two chitinases, Ym1 and Chia protein (an acidic mammalian chitinase). The increases were statistically significant in WT mice, but did not achieve significance in the SP-A-/- mice. These proteins appear to play a role in asthma, chronic obstructive pulmonary disease, and other inflammatory lung diseases [37-39] although their exact action in these conditions is not known. Annexin A1 had a similar pattern, increasing with infection in both strains, but only reaching significance in the WT mice. The basis for this increase is unclear but its altered expression here may play a protective role against infection. This postulate is consistent with various reported actions of Annexin A1. These include its protective role after organ injury [40] that could be similar to the case with infection, and its ability to promote apoptosis and limit cell proliferation [41], a potentially important function in the present situation given the rapid influx of immune cells following lung infection. Also increased was the Clara cell protein, CC10 or CC16, which is considered to be a marker of lung injury [42] and may have anti-inflammatory activity. Pronounced increases in its levels of expression were seen in both strains, but were greater in the SP-A-/- mice. These increases appear to be a response to the lung injury resulting from *K. pneumoniae *infection.

We also observed increases in alpha and beta chains of hemoglobin in both strains. Unlike the changes described above, the degree of change for both proteins was greater in the SP-A-/- mice than in WT. It should be pointed out that we did not observe corresponding increases in other serum proteins including albumin, ceruloplasmin, and haptoglobin indicating that these increases in hemoglobin are probably not due to serum protein leakage into the alveolar spaces. Hemoglobin expression has been shown to occur in alveolar epithelial cells [43]. Moreover, it has been shown that cell-free hemoglobin may play a role in the scavenging of NO [44], the levels of which increase in response to various stresses. Thus, the function of this hemoglobin produced in the lung may be one of protection against nitrosative stress as postulated previously [43,44]. This protection may come about via its ability to bind NO and thereby reduce harmful reactive species such as peroxynitrite [43,44].

The above changes describe aspects of the response to infection that were similar in both mouse strains, although in many cases (30 of 42) the magnitude of these changes was greater in the WT mice than in SP-A-/- mice.

##### b) Responses that differ between strains

In addition to the list of similar responses described above, there were changes occurring between 4 and 24 hr that followed different patterns (i.e. increased in WT; decreased in SP-A-/- or vice versa Table 4). These included annexin A5, apolipoprotein A-1, and lysozyme. Apolipoprotein A-1 (Apo A-1) is a known negative acute phase protein and during infection in the WT mice its levels decreased by 166%. Among other actions, Apo A-1 neutralizes LPS and has anti-inflammatory activity [35,45]. The decrease in its levels as the duration of infection increases in WT mice (thereby lessening its anti-inflammatory influence), could be interpreted as permitting a vigorous defense against the infecting organism. During the same interval in the SP-A-/- mice, levels of Apo A-1 increase by 38%. Given the actions of Apo A-1, this increase could impede the response against the *K. pneumoniae *infection: a) by neutralizing the effects of LPS and thereby the response to it; and b) by limiting the extent of the inflammatory response to infection, thus compromising defenses against the infecting pathogen. The basis for these differences and their significance remain to be determined. We have shown in previously published work [12] that the clinical course of WT and SP-A-/- mice differs significantly in the pneumonia model, with the SP-A-/- mice being more susceptible.

We speculate that SP-A functions indirectly as a regulator of lung host defense function, possibly via the alveolar macrophage, resulting in lower baseline levels of many DEF proteins in SP-A-/- mice. These deficits in the SP-A-/- mice appear to be rapidly compensated for with the induction of infection, but without a corresponding improvement in clinical status (i.e. survival). This lack of improvement indicates that the direct effects of SP-A on host defense against *K. pneumoniae *(i.e. enhancement of phagocytosis [12,15] or bacterial killing) may be more instrumental in determining the clinical course of the infection.

## Conclusion

In summary, this proteomic comparison of BAL proteins in WT and SP-A-/- mice under normal conditions and after infection with *K. pneumoniae *provide us with the following information. 1) Proteins involved in the regulation of host defense and redox balance are reduced in the SP-A-/- mice under baseline conditions. Prior evidence that the alveolar macrophages of SP-A-/- mice are dysfunctional or hypoactive [12] lead us to speculate that reduced levels of regulatory molecules produced by these macrophages may be responsible for these lower levels of expression of DEF and RED proteins. 2) Although SP-A-/- mice have reduced levels of DEF and RED proteins under baseline conditions, when faced with an appropriate stimulus (in this case infection with *K. pneumoniae*) they respond vigorously by increasing the levels of many of the molecules that had been at low levels. 3) Despite baseline differences between mouse strains, the pattern (but not necessarily the magnitude) of changes in protein expression in response to infection in many respects is similar in both strains. 4) However, there are a few conspicuous differences in the regulatory patterns of some proteins after infection. These observations demonstrate that SP-A may directly or indirectly alter the regulation of a number of proteins involved in host defense.

Based on this study and previously published work [12,15] we propose a model in which SP-A regulates host defense function by both indirect and direct mechanisms. The indirect effects, as documented in this study, comprise a broad-based regulation of levels of expression of a number of BAL proteins important for innate immunity and host defense function. In the *K. pneumoniae *model employed here these deficits were rapidly reversed by the infectious challenge, perhaps indicating overlap in the regulation of various mediators by SP-A and bacterial components. However, despite this vigorous response, the SP-A-/- mice have increased mortality and morbidity when compared to WT mice [12,15], suggesting that, at least with respect to *K. pneumoniae*, direct effects of SP-A on host defense function (i.e. enhancement of phagocytosis) are more important than the pleiotropic indirect effects on the BAL proteome.

## Competing interests

The authors declare that they have no competing interests.

## Authors' contributions

MA infected the animals, collected samples, ran gels, did preliminary analysis, and assisted with the writing of the manuscript. TMU organized and analyzed data, and participated in the writing of the manuscript. RH assisted with animal work and gels and helped with preliminary analysis and assisted with writing. ANM assisted with bacterial cultures, preparation, and infection. WMF did most of the MALDI-ToF/ToF analysis and assisted with evaluation of mass spec data. JF assisted with study design and data interpretation and participated in manuscript preparation. DSP designed the study, interpreted data, and prepared the manuscript. All authors read and approved the final manuscript.

## Supplementary Material

Additional file 1 **MIAPE: Gel Electrophoresis**. File containing Minimum information about a proteomics experiment - Gel Electrophoresis in the format recommended by the Human Proteome Organization Proteomic Standards Initiative. Click here for file

Additional file 2 **MIAPE: Gel Informatics**. File containing Minimum information about a proteomics experiment - Gel Informatics in the format recommended by the Human Proteome Organization Proteomic Standards Initiative. Click here for file
