# Supplementary material for: Differences in the BAL proteome after Klebsiella pneumoniae infection in wild type and SP-A-/- mice
Source: Proteome Sci. 2010 Jun 17;8:34. doi: 10.1186/1477-5956-8-34 (PMC2911411; doi:10.1186/1477-5956-8-34)
Supplement: Additional file 2 — MIAPE: Gel Informatics. File containing Minimum information about a proteomics experiment - Gel Informatics in the format recommended by the Human Proteome Organization Proteomic Standards Initiative. [file 1477-5956-8-34-S2.DOC]

Additional File 2

MIAPE: Gel Informatics

*Version 1.0, July, 2009*

Reporting requirements for gel informatics data

1. General features
   1. Date stamp: 2008-05-13
   2. Responsible person or institutional role: Mehboob Ali, Post-Doctoral Fellow, Penn State Center for Host defense, Inflammation, and Lung Disease (CHILD) Research, Department of Pediatrics, P.O. Box 850, Hershey, PA 17033
   3. Electrophoresis type: 2D-DIGE with PAGE electrophoresis
   4. Electrophoresis context: Differences in the BAL proteome after *Klebsiella pneumoniae* infection in wild type and SP-A-/- mice
   5. Image(s): Available upon request
   6. Image analysis software: Progenesis SameSpots v2.0 (Nonlinear Dynamics)
   7. Statistical analysis software: Progenesis SameSpots v2.0 (Nonlinear Dynamics), Excel (Microsoft)
2. Gel analysis design
   1. Type: Directed
   2. Replicates: n=4 per group
   3. Groups: 6 groups (wild type control, wild type exposed to K. pneumoniae for 4 hr, wild type exposed to K. pneumoniae for 24 hr, SP-A knockout control, SP-A knockout exposed to K. pneumoniae for 4 hr, and SP-A knockout exposed to K. pneumoniae for 24 hr)
   4. Internal standard: Cy2 normalization pool of equal amount of protein from all study samples run on each analytical gel
   5. External standard: Cy3/Cy5 counterbalancing to eliminate dye-based artifacts
3. Image preparation
   1. Software: ImageQuant TL (GE)
   2. Preparation steps: Images obtained using the Typhoon 9400 Variable Mode Imager (GE Healthcare) in the GEL file format (.gel) and cropped using ImageQuant TL (GE)
      1. Analytical (quantitative) gels: Laser voltages were optimized for each fluorophore prior to scanning to avoid signal saturation. Identical laser settings were then used to scan each gel
      2. Preparative/picking gels: Fixed with ethanol/acetic acid and post-stained with Deep Purple Total Protein Stain (GE), scanned independently from analytical gels
      3. All gels were scanned at 100μm resolution
      4. See MIAPE Gel Electrophoresis supplement for more specific details of image collection
4. Image analysis pre-processing
   1. Input image(s): Images obtained using the Typhoon 9400 Variable Mode Imager (GE) in the GEL file format (.gel) and cropped using ImageQuant TL (GE)
   2. Software***:*** ImageQuant TL (GE)
   3. Processing steps: See image preparation above
5. Data extraction process
   1. Input image(s): Images obtained using the Typhoon 9400 Variable Mode Imager (GE) in the GEL file format (.gel) and cropped using ImageQuant TL (GE) and are available upon request
   2. Image quality control: Image QC done using Progenesis SameSpots v2.0 (Nonlinear Dynamics) to check images for bit depth, color, manipulation prior to analysis, proper file type, saturation, low dynamic range, and stretched contrast
   3. Image alignment
      1. Automatic gel alignment using Progenesis SameSpots v2.0 (Nonlinear Dynamics) to allow for more accurate spot matching
      2. Gel alignment manually edited following automated alignment protocols
   4. Feature detection
      1. Automatic spot detection using Progenesis SameSpots v2.0 (Nonlinear Dynamics)
      2. Features were manually edited following automated spot detection protocols
   5. Matching
      1. Algorithm: Progenesis SameSpots v2.0 (Nonlinear Dynamics)
      2. Reference image(s) used: 89910 Standard Cy2 aligned for IPG 4-7 and 31847 Standard Cy2 aligned for IPG 7-11NL
      3. Landmarks: Vectors were automatically and manually placed
      4. Match editing: Automatic spot matching using Progenesis SameSpots v2.0 (Nonlinear Dynamics) followed by manual edited to confirm matches
      5. One-hundred percent spot matching across all gels without missing values was set as a requirement for spot inclusion for data analysis
   6. Feature quantitation
      1. Type: Normalized Volume
      2. Quantitation: Progenesis SameSpots v2.0 (Nonlinear Dynamics)
      3. Background subtraction: N/A
      4. Normalization: Progenesis SameSpots v2.0 (Nonlinear Dynamics)
6. Data analysis
   1. Analysis intent: Features with ANOVA (p<0.05) and/or t-test (p<0.05)
   2. Software: Progenesis SameSpots v2.0 (Nonlinear Dynamics), Excel (Microsoft)
   3. Type: ANOVA with false-discovery rates based on Progenesis assigned q-value, t-test, principal component analysis (PCA)
   4. Parameters: Not blinded
   5. Input data: Normalized volume (Cy3/Cy2 and Cy5/Cy2)
7. Data reporting
   1. List of image features: Excel file available upon request
   2. List of matches: Excel file available upon request
   3. Description of analysis results: Excel file available upon request
